# Pneumothorax spontané de l’enfant

**DOI:** 10.11604/pamj.2020.35.24.8588

**Published:** 2020-02-03

**Authors:** Abdelhalim Mahmoudi, Youssef Bouabdallah

**Affiliations:** 1 Service de Chirurgie Pédiatrique, CHU Hassan II, Faculté de Médecine et de Pharmacie, Université Sidi Mohamed Ben Abdellah, Fès, Maroc

**Keywords:** Pneumothorax, malformation bulleuse, enfant, Pneumothorax, bullous disorders, child

## Image in medicine

Spontaneous pneumothorax is a rare pathology in children, which can lead to life-threatening complications. We here report a rare cause of spontaneous pneumothorax such as bullous disorders. The study included a 13-year old girl with a year-history of recurrent bronchopneumopathies. Symptoms were aggravated by the occurrence of stage III dyspnoea (according to the New York Heart Association (NYHA)). Clinical examination performed at the time of admission showed conscious patient, polypneic at 35 breaths/min, 90% ambient air saturation and 99% ambient air saturation under 2 liters of O2. Pleuropulmonary examination showed syndrome of right aerial effusion. Chest x-ray was performed which showed right pneumothorax. The patient underwent chest drainage and, given the persistence of pneumothorax, chest CT scan was performed which showed the presence of a bleb at the level of the upper lobe of the right lung. Then the patient underwent thoracoscopy with resection of the blebs and talcage. Patient’s outcome had a median 3-year follow-up with no recurrences.

**Figure 1:**
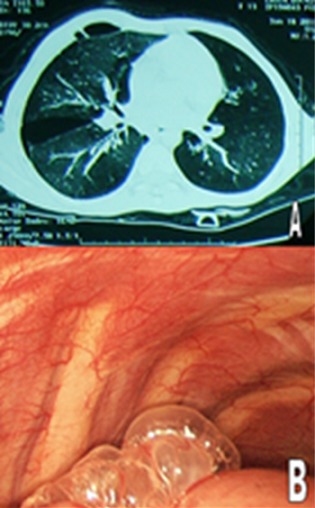
A) TDM thoracique en fenêtre parenchymateuse: bleb au niveau du lobe supérieur du poumon droit; B) exploration par thorascopie: présence d’une bleb avec réaction inflammatoire de la paroi

Le pneumothorax spontané de l’enfant est une pathologie rare, qui peut mettre en jeu le pronostic vital. A travers cette observation, on rapporte une cause rare du pneumothorax spontané qui est les malformations bulleuses. Il s’agit d’une fille de 13 ans, qui présente des broncho-pneumopathies à répétition depuis un an, la symptomatologie s’est aggravée par l’apparition d’une dyspnée stade III de la New York Heart Association (NYHA). L’examen clinique à l’admission trouve une patiente consciente polypnéique à 30 cycles/min, la saturation à l’air ambiant est de 90% et sous 2 litres d’O_2_ est de 99%. L’examen pleuro-pulmonaire trouve un syndrome d’épanchement aérien droit. Une radiographie du thorax a été faite et a montré un pneumothorax droit. La patiente a bénéficié d’un drainage thoracique, et devant la persistance du pneumothorax une TDM thoracique a été réalisée ce qui a objectivé la présence d’un bleb au niveau du lobe supérieur du poumon droit. La patiente par la suite a bénéficié d’une thorascopie avec ablation des blebs et un talcage. L’évolution était sans récidive avec un recul de 3 ans.

